# Based on the multi-scale information sharing network of fine-grained attention for agricultural pest detection

**DOI:** 10.1371/journal.pone.0286732

**Published:** 2023-10-05

**Authors:** Wang Linfeng, Liu Yong, Liu Jiayao, Wang Yunsheng, Xu Shipu

**Affiliations:** Institute of Agricultural Information Science and Technology, Shanghai Academy of Agricultural Sciences, Shanghai, China; Jeonbuk National University, REPUBLIC OF KOREA

## Abstract

It is of great significance to identify the pest species accurately and control it effectively to reduce the loss of agricultural products. The research results of this project will provide theoretical basis for preventing and controlling the spread of pests and reducing the loss of agricultural products, and have important practical significance for improving the quality of agricultural products and increasing the output of agricultural products. At the same time, it provides a kind of effective prevention and control measures for farmers, so as to ensure the safety and health of crops. Because of the slow speed and high cost of manual identification, it is necessary to establish a set of automatic pest identification system. The traditional image-based insect classifier is mainly realized by machine vision technology, but because of its high complexity, the classification efficiency is low and it is difficult to meet the needs of applications. Therefore, it is necessary to develop a new automatic insect recognition system to improve the accuracy of insect classification. There are many species and forms of insects, and the field living environment is complex. The morphological similarity between species is high, which brings difficulties to the classification of insects. In recent years, with the rapid development of deep learning technology, using artificial neural network to classify pests is an important method to establish a fast and accurate classification model. In this work, we propose a novel convolutional neural network-based model (MSSN), which includes attention mechanism, feature pyramid, and fine-grained model. The model has good scalability, can better capture the semantic information in the image, and achieve more accurate classification. We evaluated our approach on a common data set: large-scale pest data set, PlantVillage benchmark data set, and evaluated model performance using a variety of evaluation indicators, namely, macro mean accuracy (MPre), macro mean recall rate (MRec), macro mean F1-score (MF1), Accuracy (Acc) and geometric mean (GM). Experimental results show that the proposed algorithm has better performance and universality ability than the existing algorithm. For example, on the data set, the maximum accuracy we obtained was 86.35%, which exceeded the corresponding technical level. The ablation experiment was conducted on the experiment itself, and the comprehensive evaluation of the complete MSSN(scale 1+2+3) was the best in various performance indexes, demonstrating the feasibility of the innovative method in this paper.

## Introduction

Today, agriculture plays an important role in many developing countries, especially in Southeast Asia. It is estimated that these enterprises account for a considerable proportion of a country’s gross domestic product and a large proportion of its employed population. Vietnam and Thailand are the world’s largest agricultural exporters. Rice is one of the most famous agricultural products. Along with coffee, cocoa, corn, fruits, vegetables and other commodities, it contributes a large portion of the local GDP. Palm oil, for example, is an important agricultural product for Indonesia and Malaysia, both members of the Association of Southeast Asian Nations. Insects are the biggest hazard in agricultural production. Crops such as rice and wheat are vulnerable to pests, which bring huge economic losses to farmers. Therefore, in order to ensure that ASEAN countries can obtain more food from agriculture every year, the identification of crop diseases and pests in the process of agricultural production plays an important role in the early prevention and control of crops and damage to crops. Manually identifying pests in large-scale farming is a time-consuming and costly undertaking. Now, with the spread of high-quality image capture devices and advances in pattern recognition by machine learning, image-based automatic pest recognition systems promise to reduce human costs and perform the task more efficiently. When classifying insects, it is difficult to extract effective features. There are many kinds and forms of insects, which bring great difficulty to their classification and recognition. In recent years, artificial features (GIST, HOG, SIFT, SURF, etc.) have been thoroughly studied. However, the artificial characteristics lack the representation of large-scale morphological changes of multiple objects [1, 2]. At the same time, the deep learning based agroforestry data processing technology also provides a new way for researchers with strong generalization performance, and effectively avoids over-reliance on artificial features. In recent years, convolutional neural network, as an effective classification method, has been regarded as an effective classification method because it can automatically extract useful features from images without human guidance. Moreover, it performs well in recognizing complex features and different objects, and has high generalization performance. In addition to the new CNN architecture, Such as Alexnet [3], DenseNets [4], EfficientNets [5], GoogleNets [6], MobileNets [7, 8], NasNets [9], ResidualNet-works(ResNets) [10], SqueezeNet [11], eometric Group Networks(VGG) [12], etc., are all hot spots in deep learning research. They have different advantages, for example Alexnet [3] can take image classification tasks to new levels; EfficientNets [5] enable the integration of multi-tier networks. GoogleNets [6] achieves great success in the image classification task; MobileNets [7, 8] can recognize small objects; NasNets [9] can realize image classification and image retrieval. ResidualNet-works(ResNets) [10] enables end-to-end prediction models; SqueezeNet [11] can implement multitasking learning; Visual Geometric Group Networks(VGG) [12] enable object recognition in a number of different areas. Their appearance not only brings new changes to the field of deep learning, but also provides new ideas for solving more problems.

The proposed fine-grained mode of simulation attention mechanism enables efficient reuse to provide a new entry point for solving a certain problem and greatly reduces the requirement for massive computing resources [13]. This project is based on the existing PlantVillage database [14, 15] and adopts the network framework of Alexnet and GoogleNets to improve the accuracy and efficiency of vegetation pest identification methods. In addition, the technique can better handle higher-dimensional data and can more efficiently leverage correlations between data. In recent years, deep neural networks have played an increasingly important role in the classification of vegetation pests in some crops, For example, apple [16], cassava [17, 18], corn [19], cucumber [20], grape [21], corn [22], mango [23], rice [24], millet [25], guava [26] and so on, which benefit from their high recognition accuracy and strong robustness. Disease identification and classification can be carried out effectively. However, current methods are mostly focused on depth and complexity, rather than learning from traditional machine learning methods to improve detection accuracy. Therefore, it is of great significance to further study the application of deep neural network in crop vegetation insect classification.Cnn-based features are widely used to classify insects in the ImageNet Large Visual Recognition Competition (ILSVRC). WU and colleagues [27] have demonstrated that CNN-based features are more efficient than hand-drawn ones in this work. Secondly, the developmental process of metamorphosis includes egg, larva, pupa, adult and so on. Also, there are strong similarities between the two species. For each category, efficient algorithms need to capture features that express a large number of morphologic variations. So far, there has been no research on insect identification. In order to solve this problem in the classification of bird species or insect models, a fine-grained image classification algorithm is adopted in this paper. Fine-grained image classification is to extract distinguishable features from the information area of objects and classify them in the form of vectors.

Considering the influence of geographical environment and changeable weather on image information, the existing mainstream image segmentation methods are not applicable. Considering the main moving position of the target and the safety protection area of the track, combined with the actual background, this paper proposes an optimization algorithm based on the fusion of recursive attention convolutional neural network and adaptive particle swarm optimization. For the information loss caused by traditional convolution in the process of image classification and region sampling, attention learning is used to recursively operate under multi-scale, and the adaptive particle swarm optimization algorithm is used to synthesize the feature information of different scales, and continuously recursively generate regional attention from rough to fine, so as to realize the localization and detection optimization of the target region. Specifically, the attention module is used to plan the target area of the image, considering the information loss generated at each scale in the process, and introducing it into the cyclic convolution, so that it can more closely aggregate the convolution information before and after, improve the target segmentation performance of the network and reduce the amount of calculation. Attention every scales module division are on a level area location and the influence of information loss, to ensure that the final output is approaching the target location information of the image. In the whole cyclic convolution process, the above output results are processed through five pooling layers to obtain a number of attention maps that feedback the clustering correlation from different scales. Then the adaptive particle swarm optimization algorithm is introduced for weight ratio, and finally the Softmax function is used to identify and classify vegetation diseases and pests.

In this study, the superiority of the proposed optimization method in the detection of vegetation pests and diseases is verified. Based on the traditional recursive attention convolutional neural network, an accurate identification method is proposed to gather the convolution information before and after, so as to perfectly segment the target from the background, and a high performance test is achieved in the dataset. In this paper, the main contribution of the summary is as follows:

1. First, we apply the CNN model with attention mechanism to create a feature extraction program focusing on insects. Since images of insects on crops often contain complex backgrounds of leaves, dust, and branches, the mechanism of attention is crucial.
2. Secondly, this project intends to use multi-scale convolutional neural network to capture insects of different sizes.
3. Third, we propose a fine-grained image classification algorithm based on multi-scale learning to solve the problem of high inter-class similarity.
4. Finally, we will adopt the "soft voting" method to integrate the above models to further improve the performance of the system.

The rest of the article is arranged this way: In the related work section, we will explore the literature that is currently available to substantiate our motives. The experimental process section describes our proposed method in detail, and the results section gives some conclusions which show the correctness of the method we have introduced. In the conclusions section, we summarize the shortcomings of this paper and make suggestions for future researchers.

## Related work

In recent years, in agricultural production, a large number of visual research work is through a certain degree of training to solve the classification problem. In the field of insect classification, Cheng et al. [28] has proposed a new method of crop feature extraction based on deep residual network. In the complex scene, the recognition accuracy of 10 kinds of insects is 98.67%, which is better than the conventional deep learning algorithm. Liu et al. [29] proposed a new method, that is, using the obvious feature extraction algorithm to determine the object of the pest, and using the deep convolutional neural network to classify the pest. The test results show that this technique can be performed with an average accuracy of 0.951 for the calculation (" mAP "). Wang et al. [30] adopted a deep convolutional neural network (Dependency Networks) based on crop pest images. They compared two alternative deep neural networks,Lenet-5,Alexnet,convolutional cores, and the number of alternative cores. The difference of test results has great influence on classification accuracy. [31] studied the classification of crop pests and analyzed their performance. At the same time, several pre-learning deep learning systems (ResNet, GooleNet, VGG-Net) were also studied. Experiments show that the proposed method is much improved compared with other pre-training methods. In [32], the author uses DC-GAN [33] to generate enhanced images for training. Another method described in [34] is feature extraction of CNN based on prototype network [35], and Euclidean operation is carried out on it. This architecture uses triples to set up loss functions. Another recent study [36] uses FSL to model small sample data. In [37], the author trained CNN to extract general plant leaf features, and combined Siamese network with triplet loss for classification calculation. In [36, 37], two authors tested their data set using PlantVillage. Currently, there is only one ideal image of vegetation with distinct background, single leaf, unshaded and continuous light in the existing FSL data set. This method includes two core contents: 1) Embedding based on generalized samples; 2) Calculation based on the distance between the sample and the query sample. In many work-learning postures embedded [38–43], simple classifiers such as nearest neighbor method and linear classifier are used for further classification. In [41], the author adopts the nearest neighbor method. MetaoptNet [44] adopted a Linear Sequencer SiamianI network [45] uses a shared feature extractor and uses the shortest distance between the queried sample and the truth value for classification. Wu et al. [27] proposed a new insect-based large-scale reference dataset (IP102). The database contains more than 75,000 categories, including 19,000 tagged target detection images. In IP102, it was tested on the basis of manual (GIST, SIFT, SURF) and CNN (ResNet, GooleNet, VGG-Net) based functions. Ren [46] proposed a new method of interlayer features of residual data based on residual information, namely, to establish a residual network based on the interlayer features of residual data. Tests on the previous version of IP102 showed improved performance. Liu et al. [47] also proposed a new block-based multi-branch fusion residual network (Dmf-ResNet) to learn multi-scale representation. The basic residue is bonded to the bottle and the residue is pressed into the residue so that the residue forms a residue of multiple branches. The output of these branches can be linked to new modules to achieve adaptive recalibration of the response so that it can be simulated. [48, 49] A multi-branch fusion residual net method based on deep learning was proposed and applied to classify pests.

However, there are great similarities and differences between multiple species. Existing research algorithms are aimed at each category and need to capture features that can express a large number of morphologies. So far, there has been no research on insect identification. In order to solve this problem in the classification of bird species or insect models, a fine-grained image classification algorithm is adopted in this paper. Fine-grained image classification is to extract distinguishable features from the information area of objects and classify them in the form of vectors.

## Experimental process

### A. Circular attention convolutional network

The basic convolutional neural network performs convolution processing on the target image with the help of convolution kernels of different scales, and extracts various feature information including edges and textures, which provides help for subsequent information analysis and target recognition. Its basic structures include input layer, volume at the grass-roots level, pooling, the whole connection layer and output layer [50]. In the convolution operation, the appropriate convolution kernel specification and the number of steps are selected to weighted sum the pixels, and the corresponding feature information is obtained. Then after pooling layer zone, processing, data dimension reduction, prevent over fitting phenomenon. After repeated volume base layer and pooling layer, image information was continuously simplified, and finally entered the fully connected layer to determine the results.

On this basis, the introduction of attention mechanism formation of recursive convolution neural network. The mechanism of attention stems from the study of human vision. In cognitive science, due to the bottleneck of information processing, humans will selectively focus on some information while ignoring other information. The mechanism is often referred to as attention mechanism. In neural networks, the attention module is usually an additional neural network that can hard select some parts of the input, or assign different weights to different parts of the input. Through the repeated training of the neural network, the weight features are strengthened, so that the computer can identify the area that needs to be focused on in each image, so as to form attention.

The proposed structure mainly includes three levels. The network structure of the three levels is the same but the parameter information is not related to each other. Each of these levels contain classification module (VGG) and regional sampling module (APN). Classification module of input image feature extraction and classification of regional sampling module based on attention to extract the characteristics of information area, and as the next level of input; Reciprocating operation under different level results output. When training data, it is a weakly supervised behavior that relies on information labels for classification and judgment, which consumes a lot of time and energy. The framework of attention region determination in the region sampling module is single, which is not suitable for processing all kinds of multi-shape feature information. It will cause the loss of image information during classification and region sampling, which will affect the classification and sampling of the next level. Based on this, this paper proposes an optimization algorithm based on the fusion of recurrent attention convolutional neural network and adaptive particle swarm optimization.

**Fig 1:**
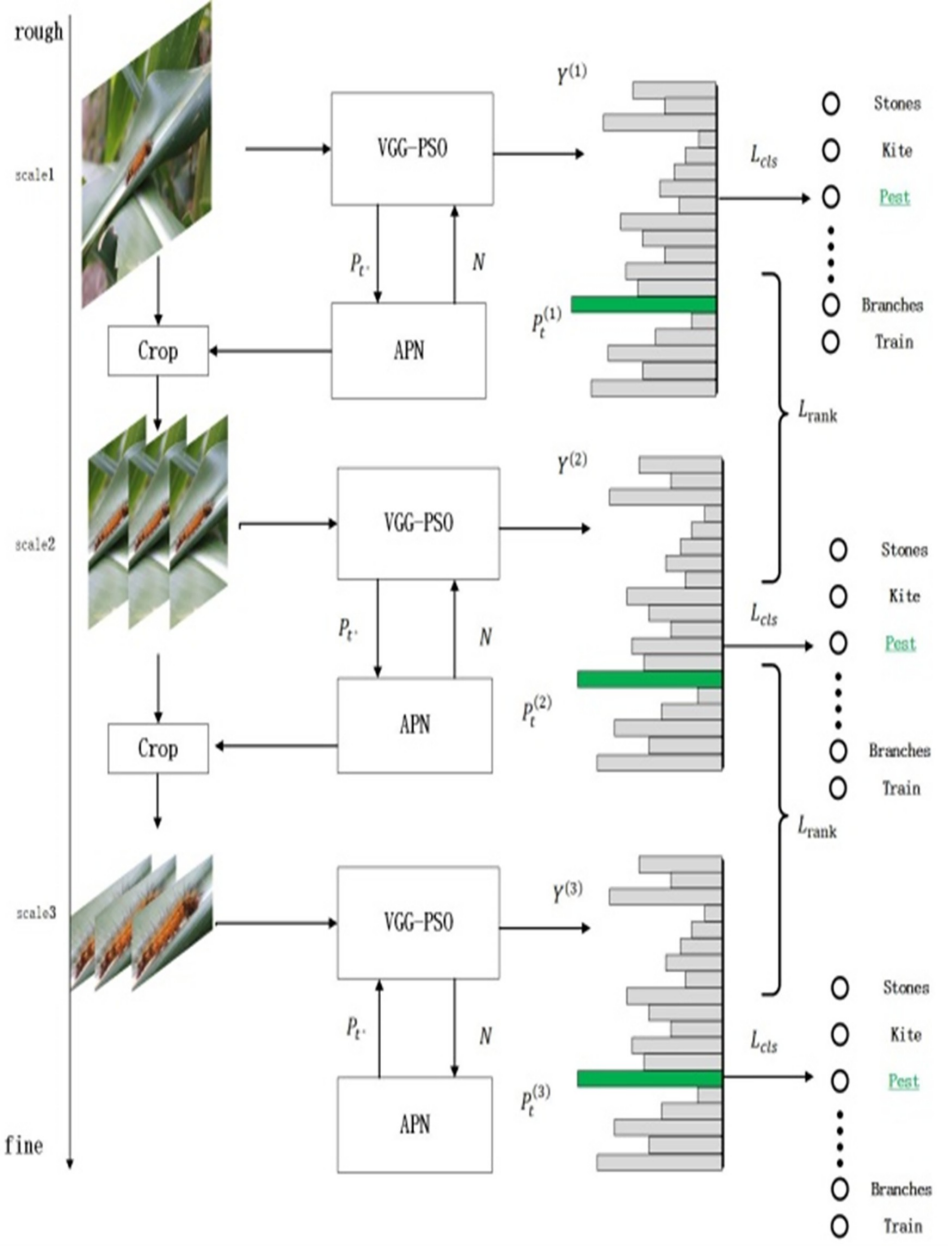
Multi-scale cascading network.

In Fig 1, a unified preprocessing operation is performed on the input image, and all the pictures of the training set and the test set are resized to the scale of 224×224. In order to make the classification information more accurate and reduce the information loss, the optimized model replaced the original classification module with the classification structure of multivariate comprehensive evaluation on the basis of maintaining the original three-layer network structure and regional sampling module. Take three-scale network structure as an example: the original input image (SCALE1) is used as the input to participate in the convolution operation of the next layer network structure (SCALE2) through one-scale image classification and feature information extraction. Reciprocating cycle three times, finally get output the results of three different scales. The specific results are shown in Fig 1:

In Fig 1, the input image is continuously classified and sampled, and the fine-grained information of the image is constantly refined. Where P_t_ is the output result after the pooling layer processing of the convolutional model. N is the sampling of attention area according to the extracted feature information, which is used as the input of classification and sampling at the next level. Y^(m)^ is the classification label of the output result at the MTH layer; $P_{t}^{\left(m\right)}$ is the probability that the classification label of the m-th layer is accurate; L_cls_ is the loss of the classification module; L_rank_ is the loss of the region sampling module.

### B. Target segmentation

The core operation of machine vision is to realize the directional segmentation of the target image, and the fully convolutional neural network [51] is the first research paper that uses convolution calculation and multi-level image segmentation. Its completely made up of multiple volumes base, there is no connection layer to implement the mapping of the input image segmentation for different specifications. Moreover, researchers use a leap-forward structure when constructing the network, so that the network can obtain high-level and low-level image features to enrich the feature information of the target image, and many famous network structures [52–54] are involved. However, full convolutional neural network also has certain defects. It cannot closely connect feature regions at different scales and limit its performance. On this basis, the relevant achievements of subsequent development are constantly exploring richer information extraction and aggregation.

The size and specification of the convolution kernel determine the range of the received information domain. Richer information requires a larger acceptance range, but it also causes an increase in computational cost and has a negative impact on the timeliness of the network structure. The convolution kernel specification [55] used in global cyclic convolution effectively saves the computational cost and the number of parameters. On the premise of not increasing the amount of calculation and the number of parameters, the extended convolution kernel can receive a wider range of information domain. Extended convolution introduces the expansion coefficient into the initial convolution kernel to determine the distance parameter between the weights in the convolution kernel. Similar methods are used in subsequent related works [56–60].

The traditional encoder-decoder structure is a common method in image segmentation. The input image is converted into a probability map of pixel categories by using the encoder-decoder structure and the connection layer, which can realize the information fusion of the front and back background at different scales. Learning deconvolution network [54] is the first image cutting using coding-decoder and achieves high performance test without using external data. SegNet [52] is also a network structure using encoder-decoder, and it proposes an innovative method of upsampling to achieve a network structure with a smaller number of parameters under the same performance. U-net [61] is widely used in the field of medicine, and many articles in this field are further based on it [62–65]. In addition, the multi-level pyramid network structure is common used methods, characteristics of the pyramid network structure [66] in the process of target detection has achieved good performance. In image segmentation, the pyramid scene parsing network [67] extracts feature information through ResNet, and then inputs the feature information into the pyramid pooling module to process feature maps of different scales and perform fusion sampling operations.

Traditional RNN is often used to solve natural language processing methods, but there are also papers that use RNN to realize the segmentation operation of image objects. With the rapid development of GAN, relevant researchers have realized various machine vision tasks, including image segmentation [68–70]. Other Angle image segmentation methods include the DecoupleSegNets [71] decouple the information feature and divide it into the main part and the edge part, and realize the optimization of the main part and the edge feature by the method of display modeling. SNE-RoadSeg [83] use a surface normals estimator, from the depth of dense information to calculate the surface normals feature space partition.

**Fig 2:**
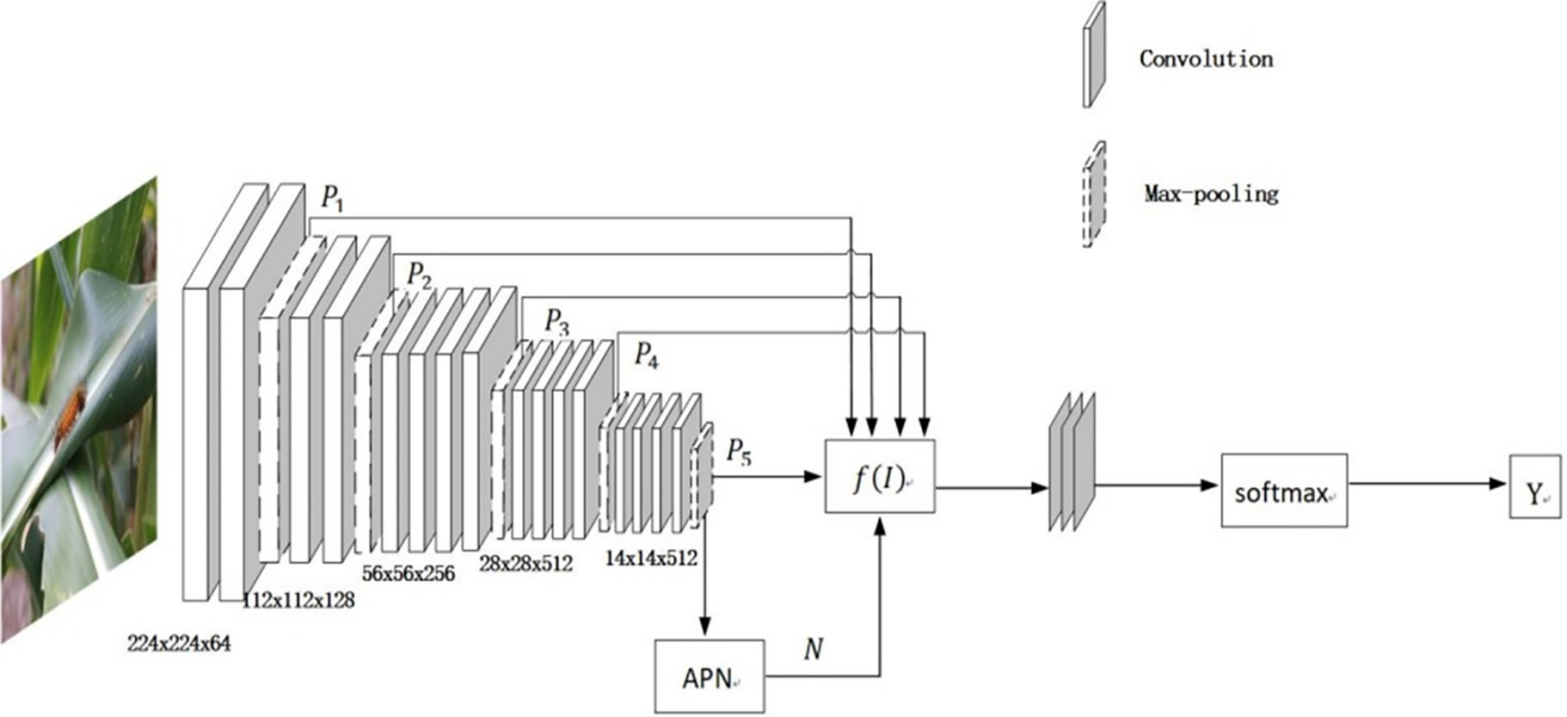
Information cascading and weight sharing between different levels.

The classification module of the traditional model usually uses the output result P_5_ of the fifth pooling layer to calculate the loss function and determine the classification label. However, the image feature information contained in P_5_ is lost due to the change of the size of the sampling module area after multi-layer convolution. Therefore, the comprehensive multivariate algorithm is introduced on the basis of the VGG module, so that the network structure can select the appropriate convolution kernel for classification, and reduce the loss of feature information caused by the classification and sampling process. The specific results are shown in Fig 2:

### C. Attention model

Recently, researchers have begun to conduct in-depth research on the attention mechanism, and the proposal of attention module has been widely used in various fields of machine vision [72–74]. Common attention modules include channel attention and spatial attention, in which channel attention enhances the network structure by strengthening the feature relation between different channels [75], and spatial attention enhances the network structure by extracting the feature information from a single pixel to a local area [76]. GANet [77] constructed a spatial gating attention module to realize the adaptive evaluation of multi-scale feature interaction mechanism. FocusNet [78] the encoding—decoder module, the attention of the parallel branch association and generate a gradient flow segmentation mask to achieve optimization. The multi-modal fusion network [79] uses multi-channel independent coding to extract feature information respectively, and designs an attention mechanism fusion module to fuse these information. GSANet [80] using selective attention extraction under different spatial location and different levels of information of pixels. Researchers [81] proposed a bilateral attention module, in which the foreground and background are respectively focused in different ways to obtain information. There is also a parallel reverse attention module [82] to solve the polyp segmentation operation of colonoscopy images, and the reverse attention module is used to realize the division of target area and boundary area. Staff [83] based on attention mechanism itself, by calculating the objective function space build focus area of each feature information.

When determining the area to be sampled on the input image, the upper left point of the image is first assumed to be the origin of the coordinate, and the right and down are set as the positive direction of the X axis and Y axis, respectively. The upper left of the area to be sampled is t_tl_ and the lower right is t_br_, and the specific Formulas (1) and (2) as follows:

$$t_{x(tl)}=t_{x}-t_{l},\;t_{y(tl)}=t_{y}-t_{l}$$ (1)

$$t_{x(br)}=t_{x}+t_{l},\;t_{y(br)}=t_{y}+t_{l}$$ (2)

After determining the area to be sampled, the element multiplication method is used to cut and enlarge the operation [84], where ⊙ represents the element multiplication, X^att^ is the area to be sampled, and M represents attention recurrence and the specific Formulas (3) and (4) as follows:

$$X^{att}=X⊙M(t_{x},t_{y},t_{l})$$ (3)

$$M=[H(x-t_{x(tl)})-H(x-t_{x(br)})]∙\;[H(y-t_{y(tl)})-H(y-t_{y(br)})]$$ (4)

In order to reduce the information loss generated in the classification module and sampling module, the derivative mapping is used to reflect the direction of attention recurrence, and the darker the color is, the more consistent it is with the direction of attention recurrence [85]. t_x_ for example, which M′(t_x_) is the focus of recursive t_x_ derivative, piecewise function is as follows (5):

$$M^{\prime }\;(t_{x})=\left\{ \begin{array}{l} <0\;x\to t_{x(tl)}\\ >0\;x\to t_{x(br)}\\ =0\;otherwise \end{array}\right.$$ (5)

**Fig 3:**
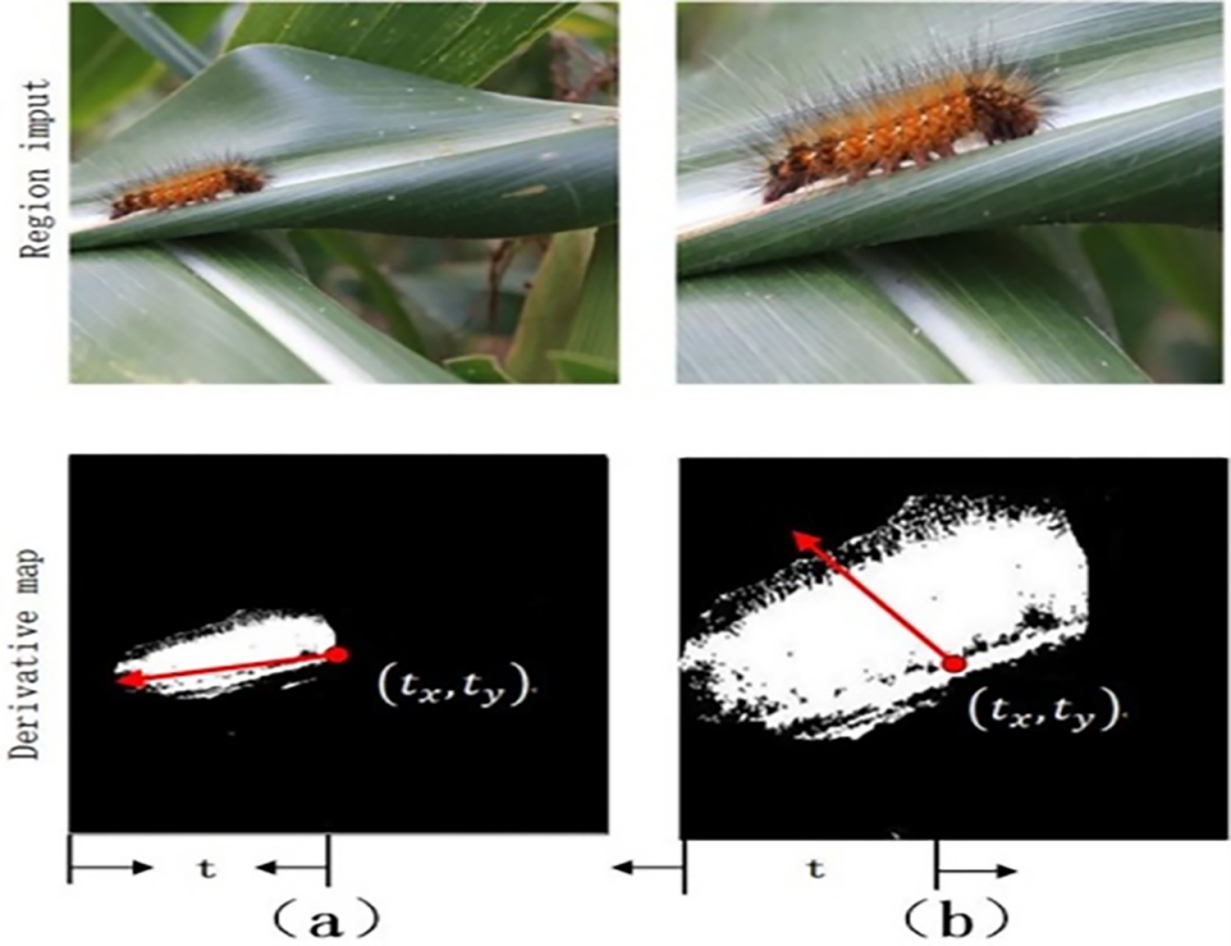
The direction of attention recursion.

Considering that the mapping result of the attention derivative is a negative transformation from the image boundary to the interior, the $L_{rank}^{\prime }(t_{x})$ result is positive, so t_x_ becomes smaller in the recursion of the lower structure, which represents the direction of human attention. The specific results are shown in Fig 3:

Based on the attention recursion network model, the label classification within the level and the region sampling between the levels will cause information loss, in which the loss function is defined as follows (6):

$$L=\sum_{m=1}^{3}\{L_{cls}(Y^{\left(m\right)},Y^{*})\}+\sum_{m=1}^{2}\left\{L_{rank}(P_{t}^{\left(m\right)},P_{t}^{\left(m+1\right)})\right\}$$ (6)

Particle swarm optimization(PSO) is a stochastic optimization algorithm, which simulates the behavior of birds flying and foraging, and makes the group achieve the optimal purpose through the collaboration between the flocks of birds. It is suitable for solving complex nonlinear optimization problems. The particle swarm optimization algorithm solves the optimization problem, and a single particle represents a feasible solution. The fitness of the particle is calculated according to the optimization objective function, and all individuals in the population are constantly moving to seek the optimal solution according to certain rules. The particle in the particle swarm moves according to the following rules (7) and (8):

$$v_{i}^{\left(t\right)}=\omega (t)v_{i}^{\left(t-1\right)}+Mr_{1}[x_{i}^{best}-x_{i}^{\left(t-1\right)}]+Lr_{2}[x^{best}-x_{i}^{\left(t-1\right)}]$$ (7)

$$x_{i}^{\left(t\right)}=x_{i}^{\left(t-1\right)}+v_{i}^{t}$$ (8)

Where, t is the time step of particle movement $(t>0);\;x_{i}^{\left(t\right)}$ is the position vector of the particle at time step t, $x_{i}^{best}$ is the historical optimal position vector of the particle in the moving process, $x_{i}^{best}$ is the historical optimal position vector of the whole population, $v_{i}^{\left(t\right)}$ is the velocity vector of the particle at time step t. M and L correspond to the attention recurrence function and information loss function, respectively. r_1_ and r_2_ are random numbers with interval [0.1]; ω(t) for adaptive inertia weight coefficient, according to the Formula is as follows (9):

$$\omega (t)=(\omega_{max}-\omega_{min})P_{s}(t)+\omega_{min}$$ (9)

Where ω_max_ and ω_min_ are the maximum and minimum values of the inertia weight, respectively, and generally the initial values are 1.0 and 0.3. P_s_(t) is the proportion of particles that move to a better position, and f_i_(x) is the fitness value of the particle at time step t, which is calculated according to the following functional Formulas (10) and (11):

$$f_{i}(x)=\frac{1}{n}\sum_{i=1}^{n}P_{s}(t)$$ (10)

$$f(I)=\omega_{1}^{c}*f_{1}+\omega_{2}^{c}*f_{2}+\cdots \omega_{5}^{c}*f_{5}$$ (11)

**Fig 4:**
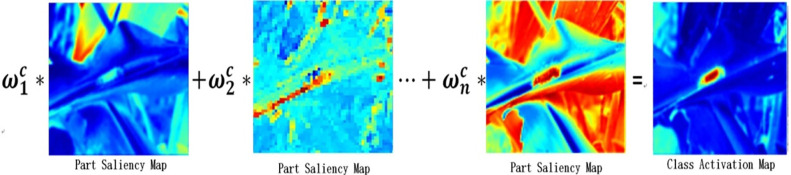
Attention weight ratio diagram.

In the convolution model, there are a total of five pooling layers to obtain five classification prediction results P_t_, in which the adaptive weights are calculated according to the recursive function of attention and the information loss function of each level, and then the target region is divided according to the five weight ratios corresponding to their respective self-fitness. Finally, the classification results of the input image are predicted by multi-level VGG-PSO labels. The classification label Y^(m)^ of each layer is put into the fully connected layer and Softmax is used to obtain the final classification result. The specific process of weighted heat map is shown in Fig 4:

### D. Data preprocessing and experimental equipment

The experiment was carried out in Windows10 environment based on Tensorflow deep learning framework. Computer configuration: Inter Core i7, 1080Ti graphics card. In learning, we use a classification based on cross entropy. Using Adam algorithm, the initial learning rate can be obtained as 5x10−5, with 16 training batches and 40–45 training cycles. When the damping is 0.96, the learning rate decreases exponentially. If, after 20 cycles, it is confirmed that the performance of the set has not improved, the training phase is over. To solve the overfitting problem, we use dropout technology with a dropate value of 0.45. Data sets including experiments are divided into training sets, verification sets and test sets in a ratio of 6:3:1. The details are shown in Table 1 below:

**Table 1:** Experimental equipment and training parameters.

name	parameter	name	parameter
DDR	128GB	size	224*224*3
CPU	Inter Core i7	iterations	40–45
GPU	1080Ti	batch	16
system	Windows10	Decay rate	0.96
editor	Pycharm 3.8	Dropout	0.45
algorithm	Adam	learning rate	5×10^−5^

The photo gallery contains 15,905 images in 10 categories. Among them, there were 1,888 palatine bugs, 973 palatine bugs, 1,759 wheat aphids, 715 noctuidae, 1,559 Oriental mole mantis, 1,269 rice planthopper, 363 rice locust bugs, 4,526 diasteridae bugs, 1,528 beet leucophora borer, and 1,325 palatine bugs. The image of the original data set is 224x224. Details are shown in Table 2 below:

**Table 2:** Shows the database in detail.

Name	Amount	Name	Amount
Trichophyta	1,888	Rice planthopper	1,269
Ricephalus oryzae	976	Rice locust	363
Wheat aphid	1,759	Diasterous bug	4,526
Noctuid moth	715	Beet leucorrhea borer	1,528
Oriental mole cricket	1,559	Marmorated bug	1,325

**Fig 5:**
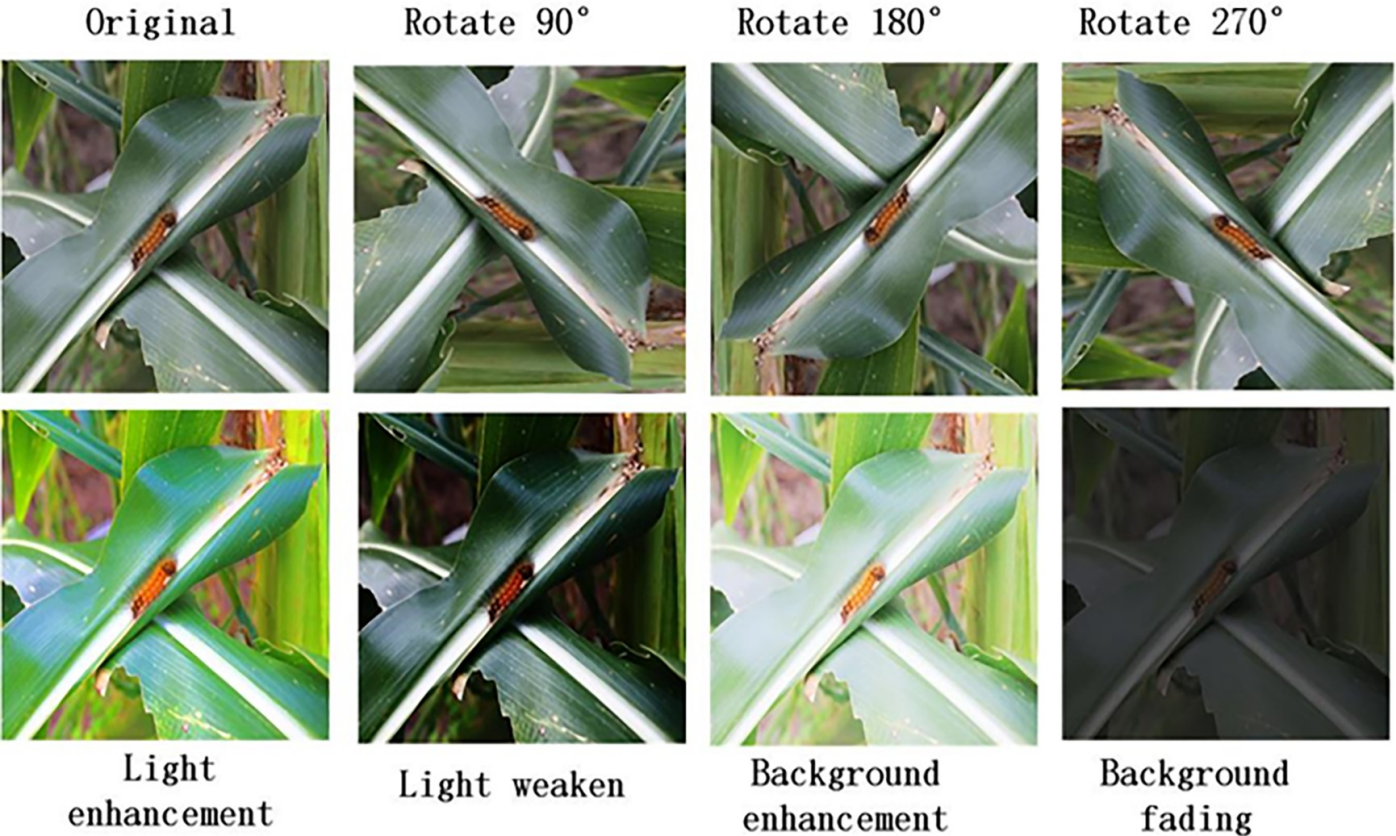
Image preprocessing.

**Fig 6:**
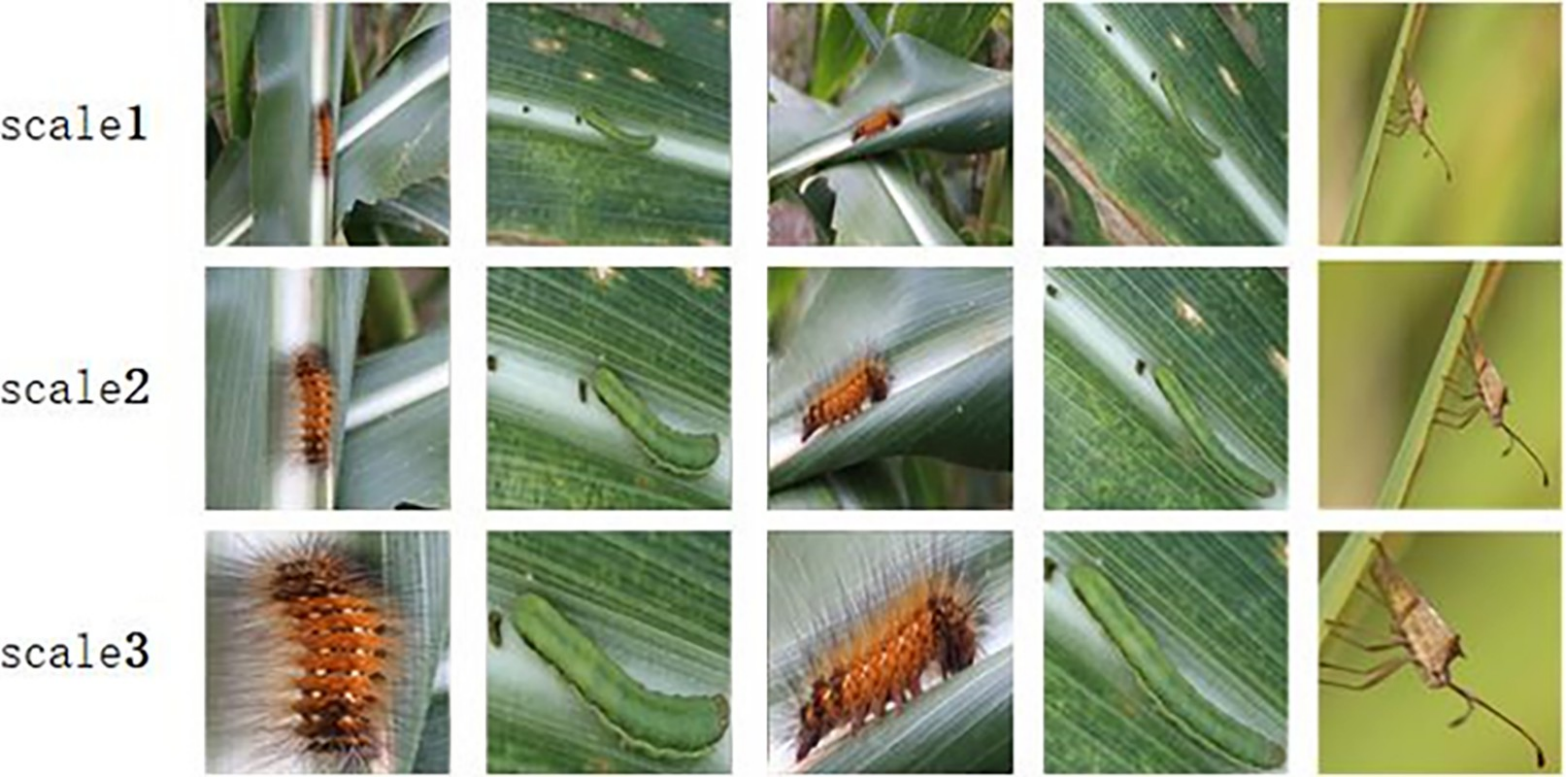
Regional magnification of different targets at three scales.

**Fig 7:**
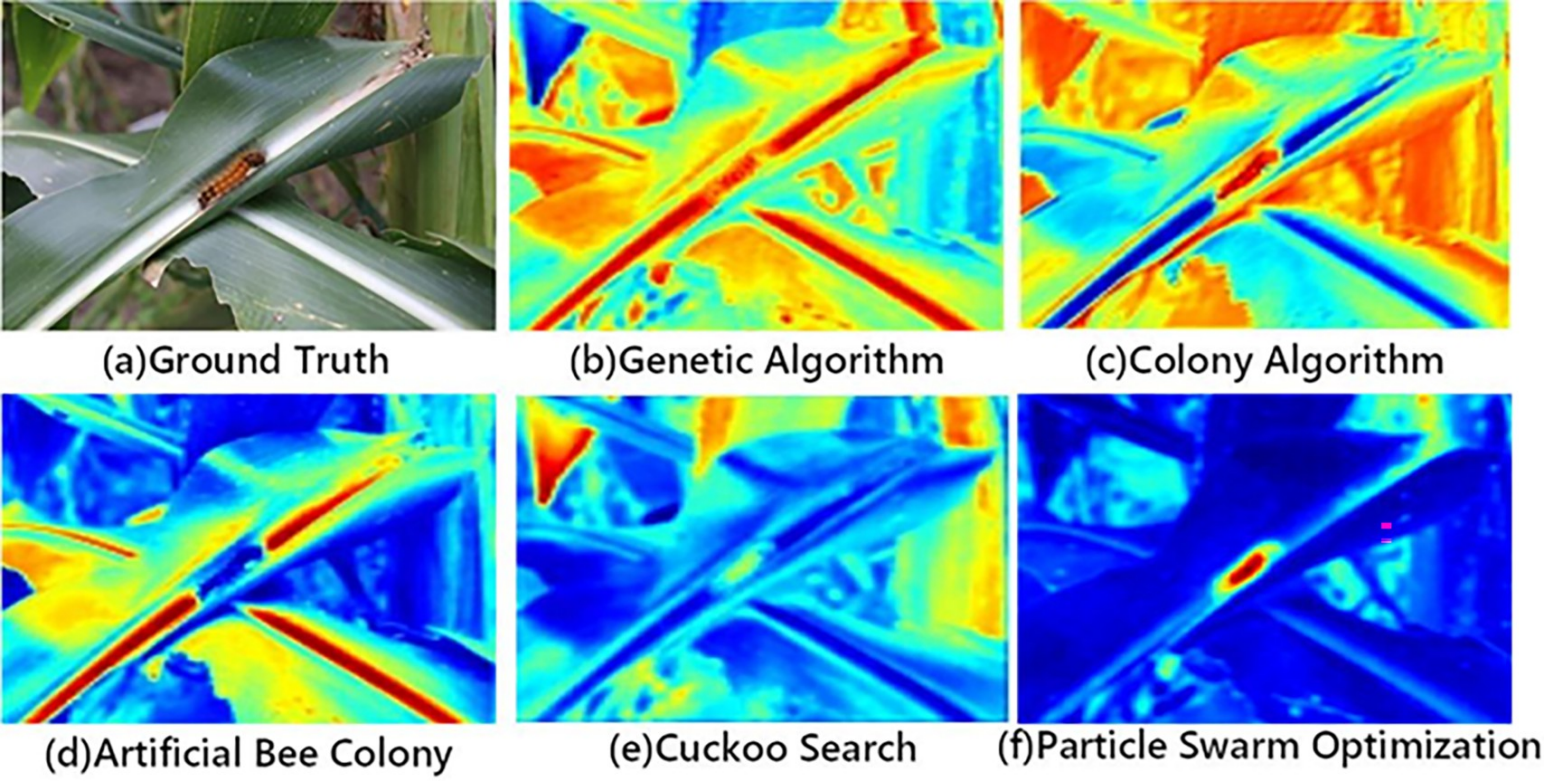
Optimization algorithm comparison.

In addition, we will add samples for each type and improve with a set of enhancement features including: randomly rotating different types of samples, mirroring (horizontal, vertical), varying brightness, etc. Details are shown in Fig 5 below:

By optimizing multi-level attention region extraction, the extracted region can not only contain the overall target structure information, but also save the local location information. It can also be clearly felt that when the input image is extracted at the second and third levels, the difference in the information it contains is more obvious, and the extracted attention area is similar to that of humans.Sense of direction is consistent, which help the exact granularity classification. As shown in Fig 6 below:

When considering the balance of information loss and clipping elements among multiple convolution layers, an adaptive optimization algorithm is needed to select the optimal solution for calculation. Genetic algorithm (GA), ant colony algorithm (ACO), particle swarm optimization (PSO), artificial bee colony algorithm (ABC), cuckoo search (CS) and so on are all based on population optimization algorithms. The probabilistic search algorithm of optimization step is implemented by iterative method. The experimental heat map obtained by the above five optimization algorithms combined with the proposed algorithm is shown in Fig 7 below:

As can be seen from Fig 7, the temperature curve of insulator identified by particle swarm optimization algorithm coincides with the actual target detection point, so particle swarm optimization algorithm is adopted in this paper. Particle swarm optimization (PSO) is a random optimization method that simulates the flight and foraging of birds. This method is suitable for solving complex nonlinear optimization problems.

### E. Evaluation index

This metric consists of macromean precision (MPre), macromean recall (MRec), macromean F1-composition score (MF1), accuracy (Acc), and geometric mean (GM).To be equally important, we calculated the recall rates for each category and then averaged them to obtain the MRec shown below (12) and (13):

$${Rec}_{c}=\frac{{TP}_{c}}{{TP}_{c}+{FN}_{c}}$$ (12)

$${MRec}_{c}=\frac{{\sum_{c=1}^{C}Rec}_{c}}{C}$$ (13)

C represents the number of a category. TP_c_ and FN_c_ denote pseudo-negative and pseudo-negative values.Also, we will carry out calculations. Pre_c_ and MPre_c_ look like this (14) and (15):

$${Pre}_{c}=\frac{{TP}_{c}}{{TP}_{c}+{FP}_{c}}$$ (14)

$${MPre}_{c}=\frac{{\sum_{c=1}^{C}Pre}_{c}}{C}$$ (15)

Class C of FPc is a false positive. MF1 is the average of MPre and MRec, such as follow (16):

$$MF1=2\frac{MPre∙MRec}{MPre+MRec}$$ (16)

Acc is computed by the true positive value among all classes as follow (17):

$$Acc=\frac{TP}{N}$$ (17)

Where N is the number of samples. GM is calculated according to the sensitivity (S_c_). S_c_ and GM are as follows (18) and (19):

$$S_{c}=\frac{{TP}_{c}}{{TP}_{c}+{FN}_{c}}$$ (18)

$$GM=\prod_{c=1}^{C}\sqrt[{c}]{S_{c}}$$ (19)

If only one S_c_ is 0, then GM is 0.To avoid this problem, we replaced the S_c_ with 0.001.

## Results

**Fig 8:**
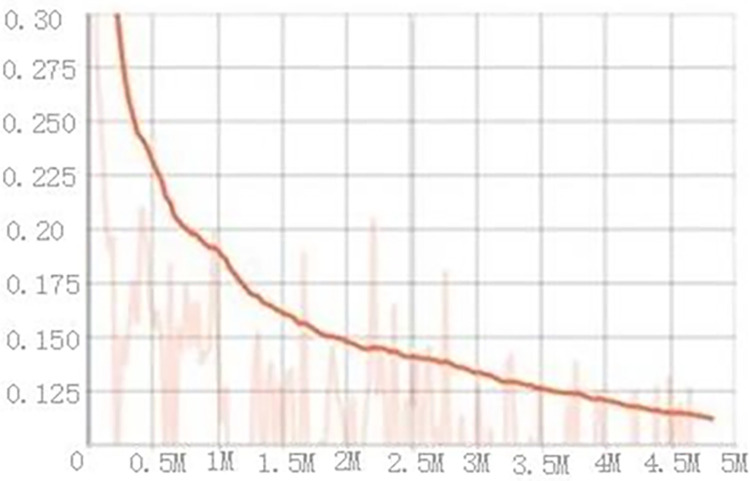
Recursive attention function and loss function change.

**Fig 9:**
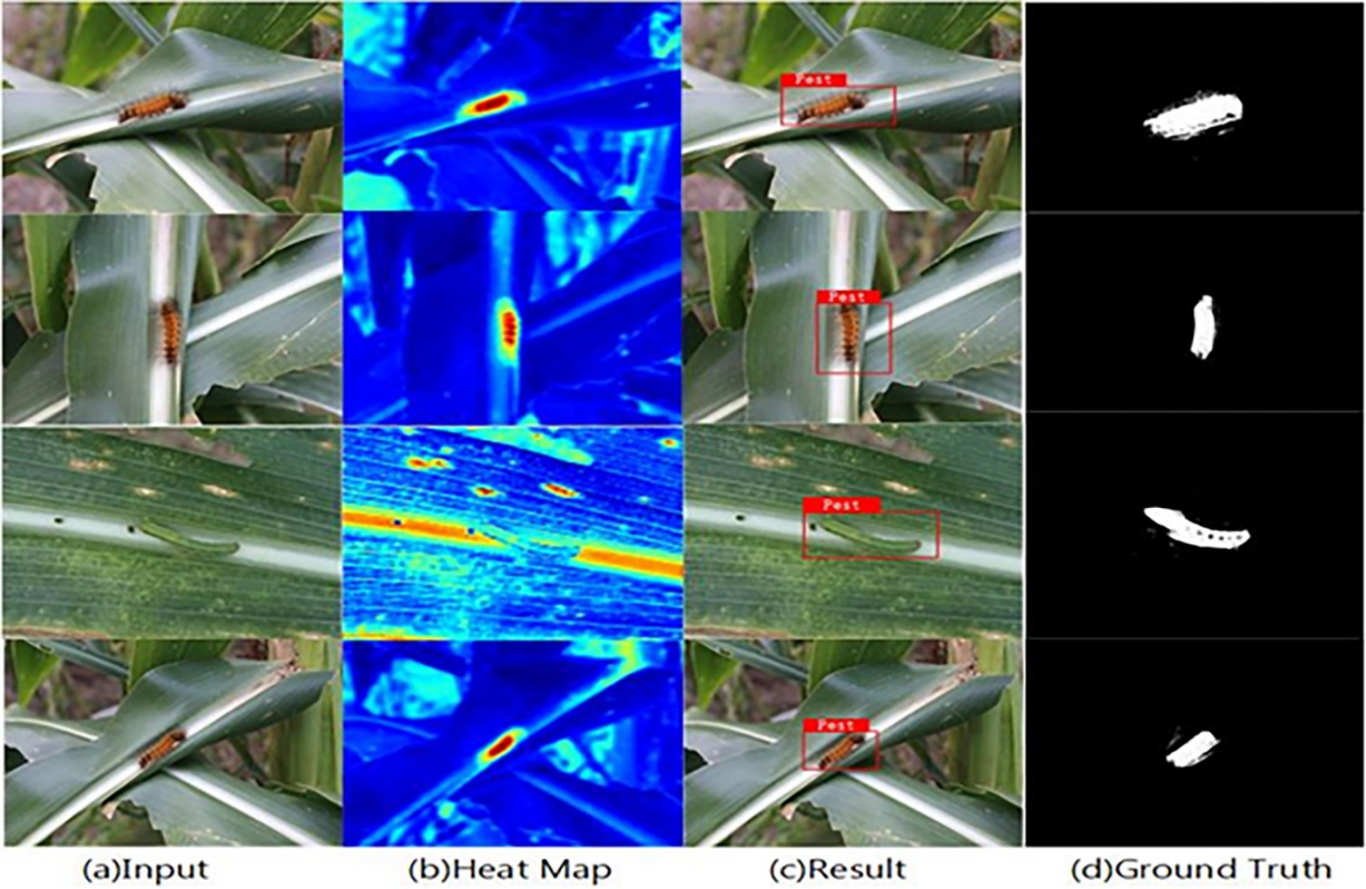
Experiment process.

It can be seen from the above figure that with the multi-level recurrent convolution operation, the deeper the network structure is, the stronger the ability to extract the feature model is. The changes of the recursive attention function and information loss function under different levels are shown in Fig 8:

In order to make the experimental effect more obvious, considering the results of target detection and recognition between different models, the classical model is introduced to compare the experimental results between different algorithms and verify the performance of the algorithm optimization algorithm. The specific results are shown in Fig 9 and Table 4:

And the detection results between different scales of the optimal model, the three layers of scales are tested by permutation and combination, and the relevant experimental results are shown in Table 3:

**Table 3:** Experimental results of different convolutional models.

Modle/Metric	Acc	MPre	MRec	MF1	GM
MSSN(scale 1)	80.99	83.09	85.91	84.09	89.90
MSSN(scale 2)	83.02	75.66	87.58	86.29	82.15
MSSN(scale 3)	80.62	82.72	84.94	83.47	89.79
MSSN(scale 1+2)	82.35	82.68	89.33	84.73	88.63
MSSN(scale 1+3)	85.35	85.92	80.94	87.85	82.72
MSSN(scale 2+3)	86.23	86.23	82.35	86.13	88.62
MSSN(scale 1+2+3)	**86.35**	**91.02**	**85.56**	**89.35**	**92.23**

As can be seen from the above table, the target detection accuracy under different scales is different. The accuracy of single-scale first layer, second layer and third layer is 80.99%,83.02% and 80.62% respectively, which is 6.02%,3.86% and 6.63% respectively compared with the best 86.35% obtained by the complete three-layer scale (scale 1+2+3) connected network model. Although there is a 0.14% difference between MSSN(scale 2+3) and MSSN(scale 1+2+3) in the detection accuracy, MSSN(scale 1+2+3) has a macro mean accuracy (MPre) of 91.02% in addition to ACC index. The macro average recall rate (MRec) was 85.56%, the macro average F1-score (MF1) was 89.35% and the geometric average (GM) was 92.23%. In terms of the overall performance evaluation index, MSSN(scale 1+2+3) had a better effect. At the same time, the performance of different algorithms is compared in the Pest dataset, as shown in Table 4:

**Table 4:** Performance comparison of different convolutional models.

Approch	Size(MB)	FLOP/s	mAP(%)	FPS	Training time(hours)
**Agarwal [86]**	15.3M	17.32	49.8	6.4	**7.5**
**Chen [87]**	26.2M	20.32	65.8	5.8	8.3
**Trivedi [88]**	27.3M	6.23	62.5	16.4	9.6
**Bhujel [89]**	25.3M	14.26	52.6	15.8	10.5
**Astani [90]**	31.2M	24.23	45.3	21.3	11.2
**MSSN (scale 1+2+3)**	**32.8M**	**4.14**	**72.3**	**26.5**	19.2

Note: Bold font indicates the value corresponding to the best performance in the current table. Abbreviation: FLOP/ S, floating-point operations per second; FPS, frames per second. MAP: mAP accuracy.

It can be seen from Table 4 that when other benchmark networks are used for comparison experiments, the indexes of 4.14FLOP/s, mAP72.3% and 26.5fps are the best, but it also results in the huge volume of the overall training network of 32.8M and the multifold increase of training time to 19.2hours.The results of agricultural pest detection on the data set achieved by the innovative algorithm proposed in this paper are shown in Table 5 below:

**Table 5:** Data sets include various pests and accuracy.

Diseased	Accuracy(%) from MSSN
Trichophyta	85.02
Ricephalus oryzae	86.11
Wheat aphid	88.64
Noctuid moth	90.40
Oriental mole cricket	92.42
Rice planthopper	93.52
Rice locust	91.31
Diasterous bug	92.04
Beet leucorrhea borer	92.71
Marmorated bug	94.52

## Conclusions

This paper proposes a recursive convolution neural network and adaptive particle swarm optimization algorithm of fusion, in view of the traditional convolution in the image classification and regional information loss during the process of sampling, through the study of attention under the multiscale recursive operations, with the help of adaptive particle swarm optimization algorithm integrated the characteristic information of different scales, from coarse to fine is recursive generation regional focus, Optimized by testing the positioning of the target area. The experimental results showed that the accuracy rate of the The test set reached 86.35%, which improved the accuracy of the detection of vegetation disease and insect pests entering the limited area. However, compared with other basic models, this method not only improves the recognition accuracy, but also reduces the memory capacity and computing speed of the model. Considering the balance between recognition accuracy and system speed is one of the future research directions of the author. In the future, this paper will further explore the diagnosis of plant diseases under the influence of various environmental factors such as image pollution, information defects and blurring, so as to ensure the safety of agricultural production.

## Supplement

**Fig 10:**
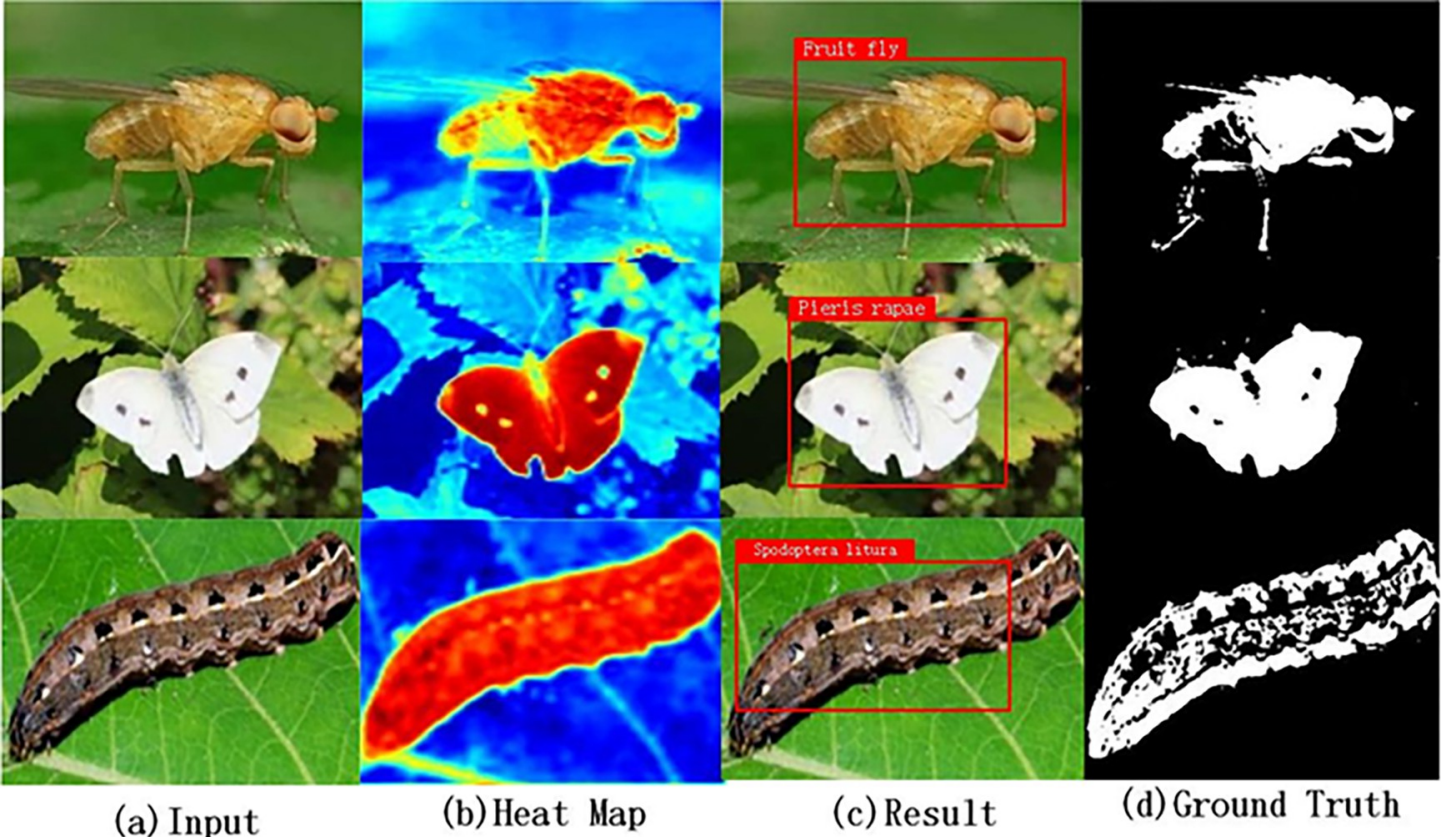
Demonstrates experimental robustness.

In the supplementary material, in addition to the pest identification study involved in Fig 9 in the main text, the detection of agricultural pests can still be achieved in the case of different sizes, body types and appearances of pests, demonstrating the generalization ability of the test method, as shown in detail in Fig 10:
